# Presenteeism and work ability: development of the Persian version of the Stanford Presenteeism Scale (P-SPS-6) and measurement of its psychometric properties

**DOI:** 10.1186/s40359-021-00617-3

**Published:** 2021-08-17

**Authors:** Fatemeh Abdi, Mehdi Jahangiri, Mojtaba Kamalinia, Rosanna Cousins, Hamidreza Mokarami

**Affiliations:** 1 Department of Occupational Health and Safety Engineering, School of Health, Shiraz University of Medical Sciences, Shiraz, Iran; 2 Department of Psychology, Liverpool Hope University, Liverpool, UK; 3 Department of Ergonomics, School of Health, Shiraz University of Medical Sciences, Shiraz, Iran

**Keywords:** Presenteeism, Absenteeism, Work ability, Job satisfaction, Emotional exhaustion, Nurses

## Abstract

**Background:**

Presenteeism is recognized in Iran’s hospitals, however little research has been done to understand and tackle the phenomenon because of a lack of valid tools to measure presenteeism. This study aimed to develop a Persian version of the Stanford Presenteeism Scale (SPS-6) and measure its psychometric properties. Another goal was to examine the relationship between presenteeism and work ability using a sample of 250 nurses.

**Methods:**

The forward–backward translation process and cross-cultural adaptation of the scale were performed according to a standard method. The psychometric properties of the scale were measured using face and content validity, construct validity based on confirmatory factor analysis (CFA), and internal consistency. Work ability score (WAS) was used to assess discriminative validity and examine the relationship between presenteeism and work ability. Measures of Job Satisfaction and Emotional Exhaustion were used to assess convergent validity with the developed presenteeism scale.

**Results:**

Mean content validity index and content validity ratio were 1 and 0.93 respectively. CFA verified the two-dimensional structure of the scale. Cronbach's alpha was 0.77. There were positive relationships between P-SPS-6 and Job Satisfaction, and P-SPS-6 and WAS. There was a significant negative relationship between P-SPS-6 and Emotional Exhaustion.

**Conclusion:**

Our findings suggested that the P-SPS-6 had appropriate psychometric properties for studying presenteeism in employees using the Persian language. Given the negative relationship between presenteeism and work ability and the negative consequences associated with it, it is necessary to regularly evaluate this stressor and to emphasize purposeful intervention programs to control or reduce it.

## Background

Managers and organizations are concerned with ways to improve job performance and reduce costs. One way to increase productivity is to minimize absenteeism [1]. However, even when employees are physically present at work, they may experience a decrease in productivity and the quality of work; a phenomenon which is called presenteeism [1–4]. Presenteeism is defined as a person being present at work, but due to health problems their usual job performance is decreased [2, 5]. Counter intuitively, attending work when one is ill—presenteeism—can be more detrimental to an organization than absenteeism, as evidenced by robust reports that presenteeism reduces productivity as much as three times more than absenteeism [6]. Similarly, the cost of presenteeism to an organization is significantly greater than for absenteeism [7]. This can explain why managers and researchers are concerned and now paying more attention to presenteeism.

Presenteeism has been associated with other negative consequences besides reduced productivity. These include reduced quality of work, job neglect, and increased errors [8–10]. Its effects in occupations such as healthcare workers dealing with vulnerable populations can be extremely serious [11, 12]. For example, nurses who work during illness may not work to a satisfactory standard to the extent of increasing risk of errors that could cause harm, or even endanger life. Similarly, those working with infection risk spreading disease to patients, visitors, and colleagues [4, 13].

The high rates of presenteeism in medical staff have been attributed to specialized roles, lack of work force, low possibility of replacement, and a strong sense of duty towards patients [11, 13, 14]. Indeed, it has recently been reported that the level of presenteeism in hospital doctors is between 53 and 86% [15]. Research has also estimated the cost of presenteeism among nurses in the United States at about $2 billion to $13 billion annually, using 2009 prices [4]. For these reasons, the majority of studies on presenteeism have targeted the healthcare sector [11, 12, 16].

Job satisfaction is an important factor in the lives of health care staff because it can affect quality of care, productivity, and performance [17, 18]. Many studies have examined the relationship between presenteeism and job satisfaction, however there is no consensus in the findings [19, 20]. Some studies have reported a positive relationship between presenteeism and job satisfaction [20, 21], while the results of other studies have indicated a negative relationship [22–24]. That is, individuals who report presenteeism, often also assess their work environment as stressful and unsatisfactory [24]. Furthermore, a longitudinal study by Baker-McClearn et al. indicates the negative association of presenteeism with job satisfaction is due to a lack of sufficient opportunity for recovery of health [22]. Therefore, going to work during times of illness and poor health can have negative consequences such as lower job satisfaction and work engagement [24].

Emotional exhaustion is another subjective variable that can occur following a decrease in employees' job satisfaction [25, 26]. Emotional exhaustion can also be the result of long-term presenteeism [27]. In this regard, research studies indicate that presenteeism leads to exhaustion and depersonalization and predicts job absence [28]. Demerouti et al. studied nurses and indicated that presenteeism was a dangerous organizational behavior that could lead to long-term burnout [29]. This was probably because presenteeism does not give individuals a chance to recover, thereby leading to emotional exhaustion in the long term [27, 29].

Another consequence of health problems can be limited physical and functional capacities and thus reduced work ability [30]. Work ability is defined as an individual's occupational competence, health, and attitude required to meet their job demands [31]. Work ability is a concept of interest to researchers because if job demands are not commensurate with the physical and mental abilities of employees, it can lead to health and safety problems, increased costs, reduced productivity, early retirement [32], turnover [33], and increased absenteeism [34]. Finding factors that affect work ability can help managers increase their employees’ work ability by controlling or modifying them [35]. There has been a lot of research in this area, and findings recognize that many factors such as age, job demands, job resources, health status, and psychological factors can affect work ability [36]. In addition, a longitudinal study by Gustafsson et al. showed a relationship between presenteeism and work ability, and that presenteeism can reduce work ability [35]. However, there has been no replication of this Swedish study in an Iranian population despite plenty of studies that have examined the relationship between sick leave and health problems [32].

The importance of presenteeism is recognized in Iran, however beyond a small qualitative study very little research has been done in this regard. One of the reasons for lack of progress is a lack of standard tools to measure presenteeism. Even though there are many tools have been developed internationally to measure presenteeism, they tend to be in English, and in that respect a barrier to use in Persian speaking populations. One of the most important and widely used tools is the Stanford Presenteeism Scale [11]. The scale was developed to measure employees' ability to concentrate and accomplish work while experiencing health problems. The 6-item version (SPS-6) of this scale was introduced by Koopman et al. [3]. Their goal was to incorporate the cognitive, emotional, and behavioral aspects of a group of employees into a practical, concise scale with excellent psychometric characteristics. The SPS-6 is short, easily applicable [11], suitable for all jobs [13] and it can evaluate the relationship between presenteeism, health problems, and employee productivity [3, 13]. To the best of our knowledge, however, there has not yet been a validated translation of this scale into Persian. Therefore, this study followed two objectives:

1. Validating and localizing the SPS-6 scale in Persian to measure presenteeism
2. Investigating the relationship between the presenteeism and the work ability among Iranian nurses

## Methods

### Design and study population

This cross-sectional survey was supported by an available sample of hospital nurses July to December 2019. The inclusion criteria were to be a qualified nurse and have at least one year of work experience. Potential participants were provided with oral information about the study and its purpose; surveys were distributed among those who gave informed consent to participate.

267 nurses joined the study. 17 participants who did not fully complete the survey were excluded providing a final sample of 250 participants. This was more than sufficient to examine the psychometric properties of the Persian SPS-6 questionnaire according to the robust quality criteria for measurement properties for health questionnaires published by Terwee et al. [37].

### Measures

The survey consisted of five sections. The first section measured demographic characteristics (sex, age, education level, work schedule, body mass index (BMI), tenure, and employment status). To ensure confidentiality and anonymity, demographic variables related to employee work (medical centers, wards, hospitals, etc.) were not collected. The other four sections were questionnaires to measure: (2) Presenteeism (SPS-6), (3) Job Satisfaction, (4) Work Ability, and (5) Emotional Exhaustion.

### Presenteeism

The SPS-6 consists of two dimensions. The first dimension is *Completing Work* which is related to the work outcomes associated with physical aspects of a job. The second dimension is *Avoiding Distraction* which is based on psychological aspects of the work process: the ability to focus on achieving work goals [11]. The items in this scale are scored on a five-point Likert scale ranging from 1 (strongly disagree) to 5 (strongly agree) with the third option (uncertain) considered a neutral score. Items 1, 3, and 4 are reverse scored. SPS-6 score is the sum of the item scores (range 6–30). According to Koopman et al. [3], a high score indicates a low level of presenteeism. In other words, the higher the score, the greater that person's ability to concentrate and finish work, despite health problems. Presenteeism was measured using a Persian version of Stanford Presenteeism Scale (P-SPS-6). The translation process is elaborated upon below.

### Job satisfaction

Job Satisfaction was measured using the average score of the three-item Job Satisfaction subscale of the Michigan Organizational Assessment Questionnaire [38]. Items were scored on a six-point Likert scale ranging from 1 (strongly disagree) to 6 (strongly agree). The psychometric properties of the Persian version of this scale were confirmed by Mokarami [39].

### Work ability

The Work Ability Score (WAS) was used to assess work ability. WAS is the first item in the Work Ability Index [31]; it considers current work ability compared with best work ability using a scale ranging from 0 (completely incapable of doing work) to 10 (fully capable of doing work). This simple and valid score has been used in many studies to evaluate work ability. The psychometric properties of WAS and its validity for assessing the work ability of Iranian employees were confirmed by Mokarami et al. [40].

### Emotional Exhaustion

Emotional Exhaustion was assessed using the 9-item subscale of the Maslach Burnout Inventory [41]. Items are scored on a seven-point Likert scale ranging from 0 (never) to 6 (very-strong). The psychometric properties of the Persian Version Maslach Burnout Inventory were confirmed by Akbari et al. [42].

### Translation and cross-cultural adaptation of P-SPS-6

To preserve the intellectual property rights of the SPS-6 scale and obtain permission to translate it, we corresponded with the developers and obtained their permission. Based on the translation and cross-cultural adaptation process proposed by Beaton et al. [43], the scale was first translated through forward translation by two translators with a good command of English.

In the second step, members of the research team, along with the two translators, compared the two Persian versions of the scale with each other and discussed and resolved inconsistencies and ambiguities. Then, each of the six items was examined in terms of its meaning, and its cross-cultural equivalence and, where necessary, revised. Finally, a single Persian provisional version of the scale was developed.

In the third step (backward translation), the Persian provisional version was sent to two other English language experts, who were not aware of the English content of the scale and were asked to back-translate it into English (backward translation). The two English versions of the scale were then reviewed and merged again by the members of the research team, and a provisional English version of the scale was obtained. This version of the scale, along with the ambiguities and disagreements, was sent to the developers of the original version for further clarification and explanation. This version was approved after making the necessary amendments.

This version was provided to 25 nurses to resolve possible ambiguities. They were interviewed in person about their understanding of the perceptions of the scale items. The data from the interviews were discussed in an expert committee, including the members of the research team, two ergonomic and occupational health specialists, and two English translators, and the required modifications were applied to the items. Lastly, the final versions were prepared for measuring the psychometric properties. Forward–backward translations of the items are presented in Table 1.

**Table 1:** Forward–Backward Translation Used in the Preparation of the Persian SPS-6

Original Items	Forward Items	Backward Items
1. Because of my (health problem)*, the stresses of my job were much harder to handle	1. در یک ماه گذشته، به‌دلیل "مشکلات سلامتی‌ام (جسمی یا روانی)"*، به‌سختی می‌توانستم از پس استرس‌های فیزیکی و روانی شغلم برآیم	1. I can cope with stress with difficulty due to "my health problems"*
2. Despite having my (health problem)*, I was able to finish hard tasks in my work	2. در یک ماه گذشته، با وجود داشتن "مشکلات سلامتی (جسمی یا روانی)"*، قادر به اتمام وظایف سخت کارم بودم	2. I could do my hard work tasks due to "my health problems"*
3. My (health problem)* distracted me from taking pleasure in my work	3. در یک ماه گذشته، "مشکلات سلامتی‌ام (جسمی یا روانی)"*، مانع از لذت بردن از کارم می‌شد	3. "My health problems"* have prevented me from enjoying working
4. I felt hopeless about finishing certain work tasks, due to my (health problem)*	4. در یک ماه گذشته، به‌دلیل "مشکلات سلامتی‌ام (جسمی یا روانی)"*، نسبت به اتمام برخی از وظایف کاری احساس ناتوانی می‌کردم	4. Due to "my health problems"*, I was desperate to complete some work tasks
5. At work, I was able to focus on achieving my goals despite my (health problem(	5. در یک ماه گذشته، باوجود "مشکلات سلامتی‌‌ام (جسمی یا روانی)"*، می‌توانستم بر روی دستیابی به اهداف کاری‌ام تمرکز کنم	5. Despite "my health problems"*, I could focus on achieving my work goals
6. Despite having my (health problem)*, I felt energetic enough to complete all my work	6. در یک ماه گذشته، باوجود داشتن "مشکلات سلامتی (جسمی یا روانی)‌"* احساس می‌کردم انرژی کافی برای اتمام همه‌کارهایم دارم	6. Despite "my health problems"*, I felt that I had enough energy for finishing all my work

*P-SPS-6*  Persian Stanford Presenteeism Scale-6

*This word can include "back pain", "cardiovascular problems", "illness", "stomach and digestive problems", "disease or a period of disease that affects mind or body" and other similar descriptions that can be replaced with the "health problems" term in each option

### Measuring validity and reliability

#### Face and content validity

A group of 30 nurses and 10 university academics (ergonomics, occupational health, and health promotion) were asked to review the face validity and qualitative content validity of the scale. They consented to supporting the study after being given an explanation of its purpose. The anonymous presenteeism questionnaires were given to the participants to evaluate the statements in terms of comprehensibility, wording, interpretation, cultural issues, and clarity. After applying the recommended minor changes, quantitative content validity—including content validity index (CVI), and content validity ratio (CVR)—was assessed.

To evaluate CVI and CVR, the 10 university academics were asked to rate the relevance and necessity of each item, respectively. According to the guidelines [44], a CVI of greater than 0.79 is suitable, between 0.7 and 0.79 requires review, and less than 0.7 is unacceptable, and the item should be removed. According to Lawshe’s table [45], items with a CVR greater than 0.62 (for ten experts) were considered necessary (p < 0.05), and the items with a lower CVR were removed.

### Construct validity

Confirmatory factor analysis (CFA) method with the maximum likelihood estimation (MLE) method was used to investigate the factorial structure of the scale. To evaluate the goodness of fit index in CFA, the root mean square error of approximation (RMSEA), the root mean square of residuals (RMR), the goodness of fit index (GFI), adjusted goodness of fit index (AGFI), the comparative fit index (CFI) and the chi-square/degrees of freedom ratio (${x}^{2}/df$) were used [46, 47]. If the CFI value is 0.95 or higher, the RMSEA is less than 0.08, the RMR is close to zero, the GFI and AGFI values ​​are 0.8 or 0.9, and the χ^2^/df is less than 3, the fit of the model is appropriate.

### Discriminative validity

To evaluate the discriminative validity, the Mann–Whitney–Wilcoxon Test was used to compare the mean P-SPS-6 scores of people with low and high work ability. WAS scores below 8 are classified as inappropriate or inadequate work ability and WAS equal to or greater than 8 is considered as the appropriate work ability [40, 48]. Here, it was assumed that mean P-SPS-6 score for people with a low work ability score would be lower than that for those with a high work ability score [2].

### Convergent validity

Convergent validity of the scale was confirmed by evaluating the relationship between P-SPS-6, Job Satisfaction, and Emotional Exhaustion scores by calculating Spearman’s correlation coefficient. Job Satisfaction and Emotional Exhaustion scores were expected to be related to the P-SPS-6 score.

### Reliability

The internal consistency of the P-SPS-6 scale was assessed using Cronbach’s alpha. A coefficient of greater than 0.7 is considered favorable [49]. Moreover, the item-to-total correlation and Cronbach’s alpha, if item deleted, were calculated separately. An item-to-total correlation greater than 0.3 for the individual scale items was considered optimal [2]. Presenteeism is a variable that depends on one's experience; it is not a consistent factor. For this reason, test–retest reliability of the scale was not evaluated in this study [3].

### Statistical analysis

All data analysis procedures were performed using IBM SPSS Statistics and AMOS software, version 23. The Kolmogorov–Smirnov test was used to test the normality of data distributions. The significance level was set at the conventional p < 0.05. Floor and ceiling effects were considered in such a way that if more than 15% of the respondents obtained the minimum (6) or maximum score (30) on the P-SPS-6 scale [50].

## Results

The mean age and work experience of the participants were 32.6 ± 3.8 years (range 22–54 years) and 8.79 ± 7.3 years (range 1–35 years), respectively. Mean BMI was 19.91 ± 3.1. The demographic characteristics of the participants are presented in Table 2. The mean and standard deviation of the P-SPS-6 items are shown in Table 3. Only 3.2% of the participants achieved the minimum score (6) and none of them achieved the maximum score (30). These results indicated the absence of floor and ceiling effects.

**Table 2:** Participants’ socio-demographic and work-related factors (N = 250)

Characteristics	N	%
*Sex*		
Male	56	22.4
Female	194	77.6
*Marital status*		
Single	103	41.2
Married	147	58.8
*Education level*		
Associate	14	5.6
Bachelor	225	90.0
MSc and above	11	4.4
*Work schedule*		
Day-work	42	16.8
Three-shift	208	83.2
*Employment status*		
Permanent	78	31.2
Temporary	172	68.8

**Table 3:** Construct validity of P-SPS-6 (N = 250)

Item	Mean (SD)	Corrected item–total correlations	Cronbach's Alpha if item deleted	Confirmatory factor analysis			
				Standardized regression weight		Critical rate	P
Q1	2.90 (1.3)	.546	.724	.688		8.727	< .001
Q2	2.62 (1.2)	.551	.723		.745	7.231	< .001
Q3	3.12 (1.3)	.519	.731	.726		8.948	< .001
Q4	2.57 (1.3)	.593	.711	.735			
Q5	2.60 (1.1)	.351	.770		.507	6.147	< .001
Q6	2.86 (1.2)	.504	.735		.657		

### Validity

Based on the results of measuring the content validity of the scale, the total CVI and CVR values ​​of the scale were 1 and 0.93, respectively, indicating the excellent content of the scale from the experts’ point of view.

**Fig. 1:**
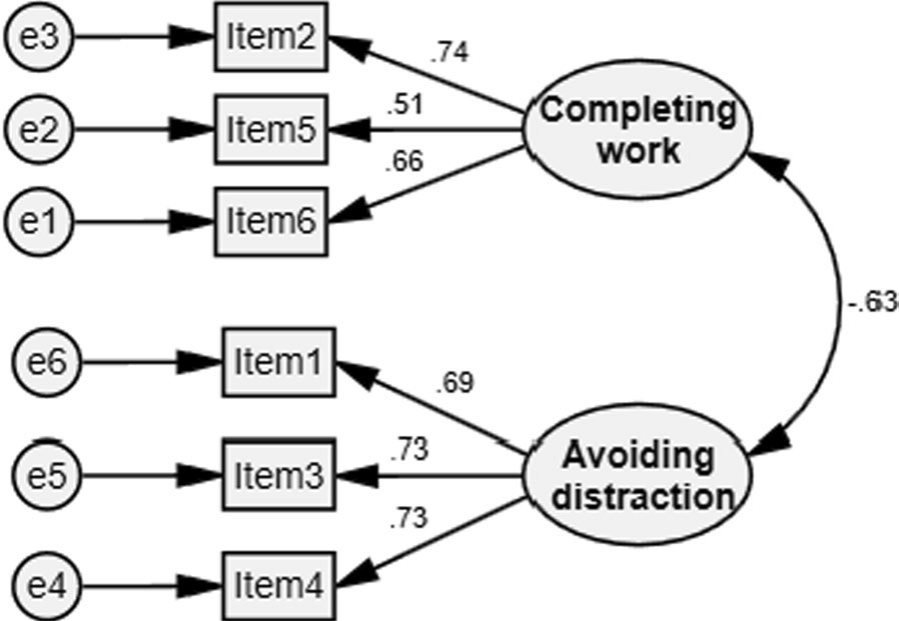
The two-factor model of the Persian version of Stanford Presenteeism Scale obtained by confirmatory factor analysis

The path diagram of the CFA of the scale with standardized factor loadings of the items is shown in Fig. 1. The goodness-of-fit indices were as follows: χ^2^ was 12.22, with eight degrees of freedom (df); χ^2^/df = 1.53. RMSEA = 0.046, RMR = 0.054, GFI = 0.98, AGFI = 0.96, and CFI = 0.99. These indicators showed a very acceptable goodness-of-fit of the model. The factor loading values of the items measuring avoiding distraction and completing were in the range of 0.69–0.73 and 0.51–0.74 (p < 0.001), indicating the most desirable factor loading of the items in both dimensions of P-SPS-6 (see Table 3).

P-SPS-6 scores were significantly lower for people with poor work ability (mean = 15.38, SD = 4.84) ​​compared with people with good work ability (mean = 19.30, SD = 4.48) (p < 0.001, Mann–Whitney-Wilcoxon Test). These results indicated good divergent validity of P-SPS-6. Moreover, there was a significant positive relationship between the P-SPS-6 and WAI scores (r = 0.42, p < 0.001). Spearman's rho analyses also showed a positive relationship between PSPS-6 score and job satisfaction score (r = 0.32, p < 0.001), and a high negative correlation between P-SPS-6 score and emotional equation score (r =−0.46, p < 0.001). These results indicated a suitable convergent validity of P-SPS-6.

### Reliability

The P-SPS-6 had good internal consistency: Cronbach's alpha = 0.77. In addition, all the items of the scale had the required consistency. Corrected item-total correlation and Cronbach's alpha if item deleted for all the P-SPS-6 items are also presented in Table 3.

## Discussion

This study aimed to develop and evaluate the psychometric properties of a new Persian version of the SPS-6 in a sample of 250 Iranian nurses. The translation and cross-cultural adaptation process of the P-SPS-6 scale was performed using a standard and valid method. The psychometric properties of the scale were confirmed based on the assessments of the face and content validity, construct validity, convergent validity, discriminative validity, and internal consistency.

The face validity and qualitative content of the P-SPS-6 were assessed by experienced nurses and ergonomic, occupational health, and health promotion specialists. Necessary amendments were made to validate the scale based on the specialists’ views. Subsequent assessment of the quantitative content validity of the scale based on CVR and CVI indicators indicated excellent content validity of the scale items.

In line with previous studies in other countries [2, 11, 13, 51], CFA showed that the new P-SPS-6 scale, like the original version, had a two-factor structure. The evaluative confirmatory factor analysis endorsed the two-dimensional structure of the questionnaire. The first factor, Completing Work, included all the positive items, and the second factor, Avoiding Distraction, included all the negative items. However, the developers specifically recommended that the sub-scales scores are not considered separately, and only the overall scale score should be used to assess presenteeism of employees [3].

Assessment of internal consistency indicated optimal reliability of P-SPS-6, and indeed the calculated Cronbach’s coefficient (0.77) was close to the coefficient calculated for the original version (0.80) [3] and in studies conducted in Italy (0.72) [13] and Portugal (0.83) [52]. To further examine the internal correlation of the scale, the item-total correlation of six items was evaluated, suggesting that all items had an acceptable correlation with the overall P-SPS-6 score.

The mean P-SPS-6 score was significantly lower among people with poor work ability compared to those who had higher work ability. This was a critical indicator of the discriminative validity of the P-SPS-6 scale. That is, the results of the present study indicated that presenteeism had a significant correlation with reduced work ability. Whilst few studies have been conducted in this area, the robust longitudinal study by Gustafsson et al. [35] showed that presenteeism could have a causative negative effect on five health outcomes, most notably the effect of repeated presenteeism on reduced work ability and physical complaints. Hockey's theory [53] of the impact of stressful factors on work performance can explain the relationship between presenteeism and work ability. Presenteeism is regarded as a form of stressor that employees choose or are required to perform. In this situation, the person is physically or mentally ill, but for some reason, they have to go to work. As a result, they need to make more compensatory efforts to stay focused on their work, or to overcome symptoms that may negatively affect their work. This can increase employee stress and anxiety [10]. There is a strong correlation between presenteeism and stress [54]. On the other hand, the higher the stress, the lower would be the capacity to pay attention to environmental stimuli, and consequently this would lower work ability [10]. Previous studies have shown that nurses who are exposed to extreme stress in the workplace experience a greater reduction in work ability than those who experience less stress [55]. The results of a study by Koopman et al. [3], using the original version of the scale, showed that the mean score of SPS-6 was significantly lower in people who reported a disability, regardless of its relation to work, compared to employees who did not report disability. Hutting et al., however, found a significant difference only between people who reported work disability compared to people who reported non-work disability or no disability [2].

The correlation between presenteeism and reduced work ability, especially in the healthcare sector, is worrying. Following previous findings [11, 56] results of the present study suggested that more than half of nurses are present at work despite being ill. Nurses go to work even when they are ill for various reasons, such as knowing their work will not be covered, not wanting to impose extra work on colleagues, feeling responsible for their patients, and challenging economic consequences [11]. Nevertheless, presenteeism in nurses leads to a decrease in physical and mental health, followed by limited physical and functional capacities, resulting in reduced work ability [30]. With increasing presenteeism and reduced work ability, the possibility of errors also increases. Errors committed by health care workers can have irreversible consequences, such as endangering their own lives or the lives of patients. Niven and Ciborowska’s [10] findings suggested that presenteeism is positively related to rates of both minor and serious errors such as giving the wrong medication or prescribing the wrong dose to patients. Therefore, it can be realizeded that interventions to reduce presenteeism are beneficial. They can help to increase the quality of work and productivity and reduce costs by maintaining and improving employees’ ability to work.

The significant relationships we found between presenteeism and job satisfaction and emotional exhaustion replicate findings from previous studies [2, 3, 28]. For instance, similar to the present study, Vandenbroeck et al. [28] reported a correlation between presenteeism and emotional exhaustion and stated that high levels of emotional exhaustion among healthcare staff could increase presenteeism. According to the conservation of resources (COR) theory [57], in occupations such as nursing with demanding job requirements, the individual is forced to use additional physical, mental, and emotional resources. Since a person's resources are limited and presenteeism leads to long-term use of resources, there is no opportunity for resources to be recovered and resources are further diminished. This can lead to increased burnout, anxiety, and reduced productivity. On the other hand, the nursing job has high emotional requirements. When too many emotional resources are consumed, it will lead to emotional exhaustion and negatively affects the treatment of patients. As a result, it may be difficult for employees to dedicate themselves to work, and thus their work energy is reduced and they experience reduced work ability [16].

## Limitations

In this study, self-reporting tools were used to consider the relationship of presenteeism and work ability. To ensure anonymity and confidentiality we could not knowingly collect information related to the workplace. It remains, however, that self-reporting tools are likely to produce biased results, even though, as Johns argues [58], it is difficult to measure presenteeism with a tool other than self-reporting instruments.

This study was performed among nurses in only one city. Therefore, caution should be exercised when interpreting and generalizing the findings regarding level of presenteeism. Future studies in Iran to assess the prevalence of presenteeism should go beyond one city and also include other occupations, as presenteeism is related to the nature of the job. A more comprehensive study of the relationship between variables such as work ability and emotional exhaustion with presenteeism is also important.

## Conclusion

The results of the present study showed that the Persian version of the SPS-6 scale has suitable psychometric characteristics and can be used in future studies as a valid and efficient tool to assess the health and productivity of Iranian employees. The P-SPS-6 whilst comprehensive, has only six items, and thus it can easily be used in a variety of workplaces, in initial screening of employees’ health, and in staff surveys. The findings of the present study showed that presenteeism, as a stressor, has a high negative correlation with work ability. The high prevalence of presenteeism among nurses can have many negative consequences, such as reduced work ability, followed by reduced quality of work and increased costs. It is necessary to evaluate this stressor continuously and to emphasize purposeful intervention programs to control or reduce it.

## Data Availability

The datasets used and/or analysed during the current study are available from the corresponding author on reasonable request.

## Abbreviations

SPS-6: Stanford Presenteeism Scale
WAI: Work Ability Score
BMI: Body Mass Index
CVR: Content Validity Ratio
CVI: Content Validity Index
CFA: Confirmatory Factor Analysis
χ2/df: Chi-Square/Degrees of Freedom Ratio
RMSEA: Root Mean Square Error of Approximation
GFI: Goodness-of-Fit Index
AGFI: Adjusted Goodness-of-Fit Index
CFI: Comparative Fit Index
